# ClusterMI: Detecting High-Order SNP Interactions Based on Clustering and Mutual Information

**DOI:** 10.3390/ijms19082267

**Published:** 2018-08-02

**Authors:** Xia Cao, Guoxian Yu, Jie Liu, Lianyin Jia, Jun Wang

**Affiliations:** 1 College of Computer and Information Science, Southwest University, Chongqing 400715, China; xiacao@email.swu.edu.cn (X.C.); gxyu@swu.edu.cn (G.Y.); jiel@email.swu.edu.cn (J.L.); 2 College of Information Engineering and Automation, Kunming University of Science and Technology, Kunming 650093, China; jlianyin@163.com

**Keywords:** genome-wide association studies, high-order SNP interactions, clustering, mutual information, improved ant colony optimization

## Abstract

Identifying single nucleotide polymorphism (SNP) interactions is considered as a popular and crucial way for explaining the missing heritability of complex diseases in genome-wide association studies (GWAS). Many approaches have been proposed to detect SNP interactions. However, existing approaches generally suffer from the high computational complexity resulting from the explosion of candidate high-order interactions. In this paper, we propose a two-stage approach (called ClusterMI) to detect high-order genome-wide SNP interactions based on significant pairwise SNP combinations. In the screening stage, to alleviate the huge computational burden, ClusterMI firstly applies a clustering algorithm combined with mutual information to divide SNPs into different clusters. Then, ClusterMI utilizes conditional mutual information to screen significant pairwise SNP combinations in each cluster. In this way, there is a higher probability of identifying significant two-locus combinations in each group, and the computational load for the follow-up search can be greatly reduced. In the search stage, two different search strategies (exhaustive search and improved ant colony optimization search) are provided to detect high-order SNP interactions based on the cardinality of significant two-locus combinations. Extensive simulation experiments show that ClusterMI has better performance than other related and competitive approaches. Experiments on two real case-control datasets from Wellcome Trust Case Control Consortium (WTCCC) also demonstrate that ClusterMI is more capable of identifying high-order SNP interactions from genome-wide data.

## 1. Introduction

Genome-wide association study (GWAS) has become a popular and powerful tool for studying human complex diseases [1]. Many GWAS methods were proposed to detect single SNPs (single nucleotide polymorphisms) over the past few years [2]. The basic principle of traditional GWAS approaches is simple and far from comprehensive. Most of them only evaluate the statistical significance of a single SNP based on the selected case and control samples. The SNP is considered to be associated with complex disease, if and only if its frequency in the cases is significantly higher or lower than that in the controls. However, single SNPs cannot completely explain the pathogenesis of human complex diseases [3,4,5]. SNP interactions among multiple genes play an essential role in the pathogenesis of complex diseases [6]. As such, precisely detecting SNP interactions contributes to better understanding of the genetic mechanisms of complex diseases.

There are two challenges in SNP interactions’ detection. The first is the intensive computational burden caused by the exponential growth of the search space. For moderate genome-wide data, even only considering pairwise SNP combinations, more than ten billion combinations need to be evaluated. The second is developing statistical tests that can effectively detect SNP interactions. To attack the first challenge, some computationally-efficient approaches have been proposed [5,7,8,9,10], such as applying filter algorithms to effectively select a subset of SNPs [11] and employing GPU for parallel computing [12]. For the second challenge, some statistical tests have been proposed for association studies, such as the chi-square test, likelihood ratio test and entropy-based test [1].

Currently, there are four categories of SNP interactions’ detection approaches: exhaustive search, stochastic search, machine learning search and stepwise search. The exhaustive search-based approaches enumerate all SNP combinations and performs the chi-square test (or logistic regression) for each combination. Ritchie et al. [13] proposed an MDR (multifactor dimensionality reduction) approach. For each interaction model, MDR partitions its genotype combinations into two classes (high-risk and low-risk) and employs cross-validation to assess the quality of each model. MDR exhaustively searches the best model with the lowest prediction error to predict the disease status. It is feasible for MDR to exhaustively search SNP interactions when the search space is small, but it is infeasible for a large dataset [7].

Stochastic search-based approaches apply random sampling procedures to search the space of SNP interactions. BEAM [14] (Bayesian epistasis association mapping) iteratively uses the Markov chain Monte Carlo (MCMC) to calculate the posterior association probability of a locus and its interaction partners with the disease. epiMODE [15] (epistatic module detection) extends BEAM; it uses Gibbs sampling and a reversible jump MCMC procedure to search for significant epistatic modules. DECMDR [16] (Differential Evolution algorithm combined with a Classification based Multifactor-Dimensionality Reduction) uses the classification-based MDR (CMDR) as a fitness measure to evaluate solutions in the differential evolution (DE) process to scan potential SNP interactions. Due to the high efficiency and simple implementation of DE, DECMDR can detect high-order SNP interactions in large genome-wide datasets.

Machine learning-based approaches build non-parametric models to detect SNP interactions, and they commonly use heuristics to avoid exhaustive search [17]. Some adopted and representative machine learning approaches are neural networks [18], random forest [19] and support vector machines [20]. When random forest is used for GWAS data, the output SNP set is defined as the most important variable set. Bureau et al. [21] extended the concept of variable importance to pairs of predictors to capture joint effects and explored the behavior of importance measures over a range of two-locus disease models. Deep learning-based approaches are also adopted in SNP interactions’ detection. Uppu et al. [22] utilized a deep feedforward neural network to classify the two-locus genotype combinations and to identify the high risk SNP interactions associated with a disease. However, for machine learning-based approaches, the computational burden associated with the search for SNP interactions is potentially huge [23], especially when searching for interactions between two or more SNPs within a GWAS dataset [7]. Besides the computational burden, the outputs may present serious challenges for biological interpretation [24].

Stepwise search approaches (mainly for high-order SNP interactions’ detection) firstly screen an SNP subset based on some low-order statistic tests, then conduct multi-locus detection based on the selected SNP subsets. EDCF [25] (epistasis detector based on the clustering of relatively frequent items) starts with two-locus interaction models, groups all genotype combinations into three clusters and then uses the chi-square test to evaluate the significance of two-locus combinations. DCHE (Dynamic Clustering for High-order genome-wide Epistatic interactions detecting) [26] dynamically groups all genotype combinations into three to six subgroups and then adopts the chi-square test to evaluate the candidate pairwise combination based on its subgroup. HiSeeker [27] employs the chi-square test and logistic regression model, which considers intermediate and significant associations with the phenotype, to select candidate pairwise SNP interactions; and then uses the exhaustive search or heuristic search to detect high-order SNP interactions from selected pairwise SNP interactions.

The aforementioned approaches have shown their capabilities in detecting high-order SNP interactions. However, there are still some limitations with these approaches. Most of these approaches detect high-order SNP interactions based on two-locus interaction models, the search space of which grows exponentially. For large genome-wide data, screening the candidate set from all possible pairwise SNP combinations is very computationally demanding.

In this paper, we propose a two-stage approach named ClusterMI (Clustering combined with Mutual Information) to detect high-order SNP interactions based on two-locus combinations. In the screening stage, considering there is a higher probability of identifying significant two-locus combinations in a high-associativity SNP group, ClusterMI firstly utilizes clustering to divide SNPs into several clusters and applies the mutual information as the association measurement. Then, ClusterMI applies conditional mutual information to screen significant pairwise SNP combinations in each cluster. This strategy avoids exhaustively analyzing all two-locus combinations and greatly reduces the search space and computation load. In the search stage, to be adaptive to different data sizes and to obtain high detection accuracy, ClusterMI provides two alternative strategies to search high-order SNP interactions. For a small candidate set, ClusterMI employs the exhaustive search. For a large candidate set, ClusterMI employs an improved ant colony optimization (ACO) algorithm [28]. Extensive experiments on simulated datasets show that ClusterMI has better performance in detecting high-order interactions than four other recently-proposed approaches: EDCF [25], DCHE [26], DECMDR [16] and HiSeeker [27]. Experiments on real Wellcome Trust Case Control Consortium (WTCCC) datasets also demonstrate that ClusterMI is more capable of identifying high-order SNP interactions from genome-wide data than these compared methods.

## 2. Results

To evaluate the performance of ClusterMI quantitatively, the power and runtime were used as the evaluation metrics. We adopt the same measurement of power suggested by Wan et al. [10]:

(1) $$Power=\frac{D_{T}}{D}$$

where $D_{T}$ is the number of datasets in which true SNP interactions can be successfully identified and *D* is the number of all datasets. Power can be seen as a measurement of accuracy in simulation experiments to compare the performance of different approaches. Since there was no training set and test set throughout the detection process, cross-validation was not suitable for ClusterMI. In actual fact, almost all SNP interaction detection approaches adopt power as the measurement. We firstly analyze the parameter sensitivity of ClusterMI in Section 2.1. Then, we perform simulation experiments and compare the performance of ClusterMI to four representative approaches in Section 2.2. Two real genome-wide datasets collected from the Wellcome Trust Case Control Consortium (WTCCC) [29] are further used to test ClusterMI in Section 2.3. All experiments were conducted on a server with Intel Xeon E5-2678, 256 GB RAM and CentOS 6.5.

### 2.1. Parameter Setting and Sensitivity Analysis

In the screening stage of ClusterMI, there were two critical parameters influencing the performance of ClusterMI: number of clusters (*k*) and cMI (conditional Mutual Information) threshold (τ), and parameter sensitivity analysis was conducted for them. Similarly, power and runtime were used to evaluate the performance on 100 simulation datasets with 4000 samples and 1000 SNPs. The parameter *k* was set from 1–8, and τ was set to 0.001, 0.005, 0.01, 0.015, 0.02, 0.025 and 0.03. By comparing the power and runtime of ClusterMI under different parameters settings, we selected the optimal input values of *k* and τ and adopted them for the simulation experiments.

**Figure 1:**
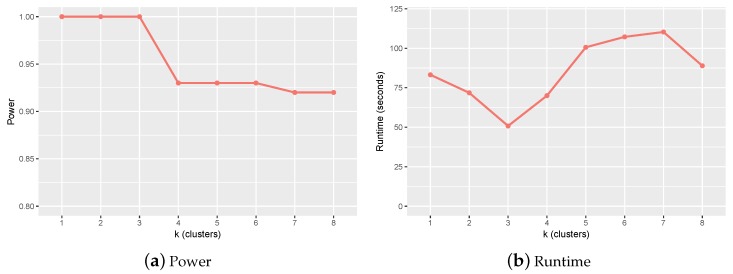
Power and runtime of ClusterMI under different numbers of clusters (*k*). (**a**) Power of ClusterMI for different *k*; (**b**) runtime of ClusterMI for different *k*.

We tested the power and runtime of ClusterMI on 100 simulation datasets, each of which contained 4000 samples and 1000 SNPs, and each dataset was generated using the simulation program in [14]. Since the data size was small, we used exhaustive search to detect SNP interactions. Figure 1 reveals the experimental results for different values of *k*. Since the increase of *k* resulted in fewer two-locus combinations in each cluster for the follow-up screen, the runtime of ClusterMI decreased from k=1 to k=3, while the power of ClusterMI remained the same. However, two SNPs decomposed from the true high-order SNP interactions were more likely divided into different clusters as k>3 and increasing, and this reduced the power. In addition, the clustering algorithm also needed more time to converge and increased the total runtime of ClusterMI as k≥3. The runtime decreased again with *k* varying from 7–8. That was because although the increased number of clusters increased the convergence time of ClusterMI, the reduced number of two-locus combinations reduced the total runtime. Thus, balancing the power and runtime of ClusterMI, we set the number of clusters to three (namely, k=3) in the simulation data. For the real dataset, considering the influence of the clustering algorithm on the total runtime of ClusterMI, we set k=11.

**Figure 2:**
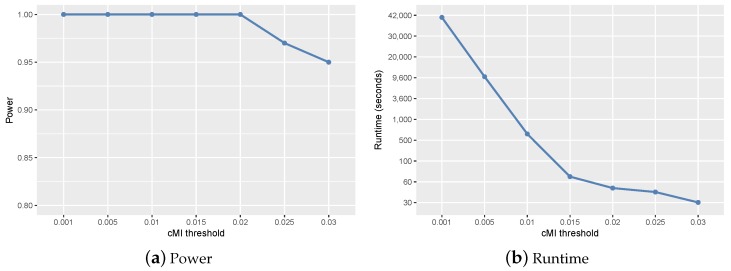
Power and runtime of ClusterMI under different cMI thresholds (*τ*). (**a**) Power of ClusterMI for different *τ*; (**b**) runtime of ClusterMI for different *τ*.

In order to find a more efficient and effective threshold, we conducted parameter sensitivity analysis for τ. We used the same simulation datasets and search strategy to test ClusterMI for different values of τ. The results are revealed in Figure 2. The runtime of ClusterMI dramatically decreased with the increase of τ, while the power of ClusterMI stayed the same with the maximum value (equal to one) until τ>0.02 and decreased with the further increase of τ. The increased τ caused fewer two-locus combinations to be retained in the candidate set *W* for high-order interactions’ detection and greatly reduced the searching cost. However, a larger threshold τ may be too conservative to retain the two-locus combinations, which were decomposed from true high-order interactions, and thus caused a reduced power. To balance the power and runtime of ClusterMI, we set the cMI threshold as τ=0.02.

In the search stage of ClusterMI, exhaustive search was used for a small ($\left|W\right|\leq 2\times 10^{3}$) candidate set, and an improved ACO search strategy was used for a large ($|W|>2\times 10^{3}$) candidate set. There were five parameters in the improved ACO search: initial pheromone value $\gamma_{i}$, the threshold $q_{0}$ to control the convergence rate, the evaporating coefficient ρ, the number of ants *n* and the maximum number of iterations MaxIter. We set these parameters according to previous studies [27,28,30].

- $\gamma_{i}$ for all two-locus combinations was set to 100.
- $q_{0}$ was set to 0.6.
- ρ ranged from 0.01–0.1 according to the size of candidate set *W*. In the simulation studies, we set ρ=0.05. In the real study, ρ was set to 0.01.
- *n* and MaxIter were determined by *W*. We set MaxIter=0.1|W| and *n* from 500–5000.

### 2.2. Experiments on Simulation Data

In the simulation data experiments, we used five three-locus disease models to compare ClusterMI with EDCF [25], DCHE [26], DECMDR [16] and HiSeeker [27]. ClusterMI had two variants: ClusterMI(A) and ClusterMI(E), where ClusterMI(A) utilized the improved ACO search strategy in the search stage to identify high-order SNP interactions, while ClusterMI(E) employed the exhaustive search strategy. Model 1, Model 2, Model 3 and Model 4 were the extension of four well-known two-locus interaction models, where Model 1 had a multiplicative effect [31], Model 2 had a threshold effect [31], Model 3 had an additive effect [14] and Model 4 had a special threshold effect [25]. Model 5 was a three-locus interaction model proposed by Zhang et al. [14]. Marginal effect size λ of a disease locus in each model was defined as [14]:

(2) $$\lambda =\frac{p_{Aa}/p_{AA}}{\left(1-p_{Aa}\right)/\left(1-p_{AA}\right)}-1$$

where $p_{AA}$ and $p_{Aa}$ denote the penetrance of genotype AA and Aa, respectively. The specification of λ was the same as the aforementioned approaches: λ=0.2 for Model 1, and λ=0.3 for Model 2, Model 3, Model 4 and Model 5. Minor allele frequencies (MAFs) were the same for the three loci at three levels: MAF = 0.1, 0.2 and 0.4; and for linkage disequilibrium (LD), we considered two scenarios: $r^{2}=1$ was simulated for directly-genotyped disease loci; $r^{2}=0.7$ was simulated for disease loci ungenotyped, but their LD markers with $r^{2}=0.7$ genotyped. We used the same simulation program as Zhang et al. [14] to simulate 100 datasets under each setting for each disease model, and this simulation protocol was also adopted by BEAM [14], SNPHarvester [32] and HiSeeker [27]. Particularly, each dataset contained 1000 SNPs, and the sample size increased from 2000–4000. The simulation program calculated the genotype of the controls and the cases according to the independence and Hardy–Weinberg equilibrium (HWE) assumption with specific disease models. In addition, it also met a specified LD structure.

**Figure 3:**
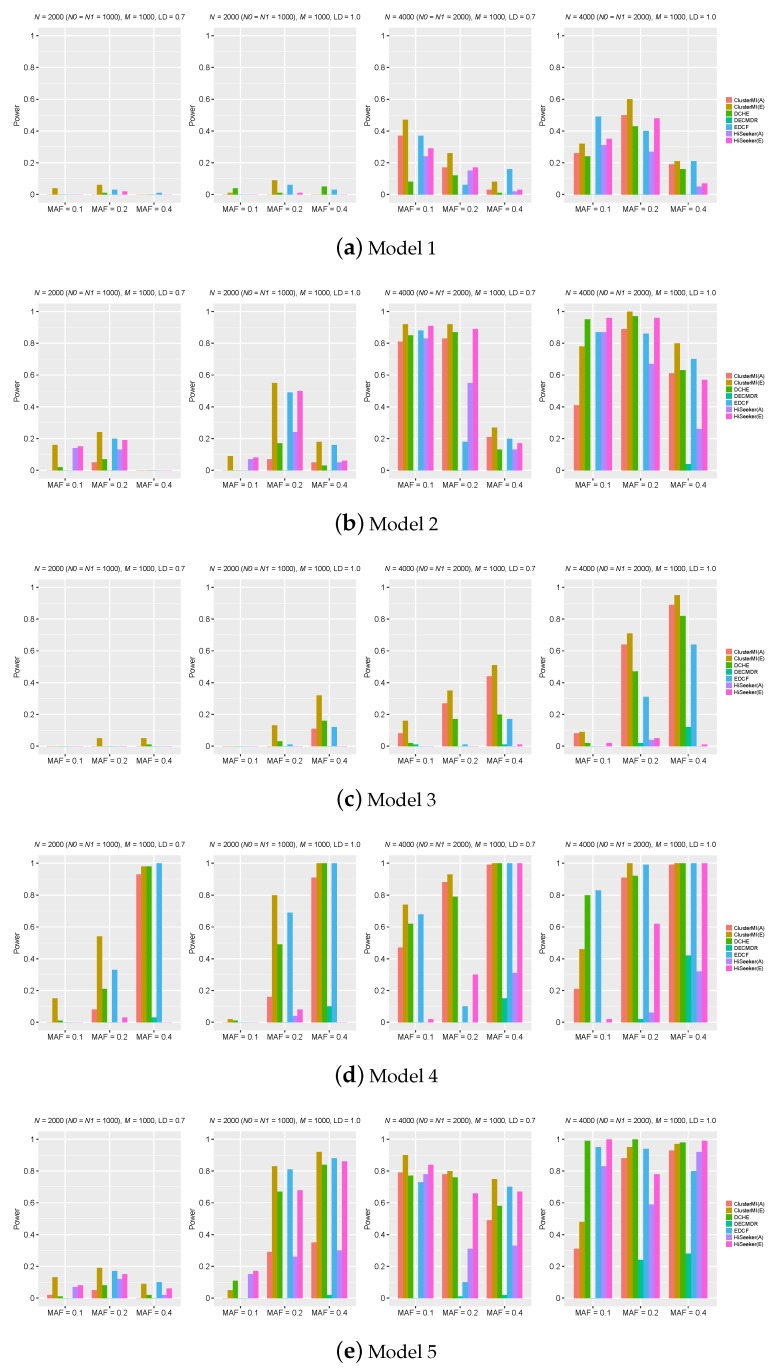
Powers of dynamic clustering for high-order genome-wide epistatic interactions detecting (DCHE), differential evolution algorithm combined with a classification based multifactordimensionality reduction (DECMDR), epistasis detector based on the clustering of relatively frequent items (EDCF), HiSeeker(A), HiSeeker(E), ClusterMI(A) and ClusterMI(E) on five three-locus disease models under different allele frequencies (MAF), sample sizes (*N*) and linkage disequilibrium (LD). *N*0 is the number of controls, *N*1 the number of cases and *M* the number of SNPs. The absence of a bar indicates no power. A: ACO search strategy, E: exhaustive search strategy. (**a**) Model 1; (**b**) Model 2; (**c**) Model 3; (**d**) Model 4; (**e**) Model 5.

Figure 3 reveals the power of different comparison approaches on the five three-locus models. ClusterMI in the first stage divided 200, 300 and 500 SNPs on average into respective clusters and about 1000 two-locus combinations for the next stage. The power of all approaches significantly improved with the sample size increasing from 2000–4000 and $r^{2}$ changing from 0.7–1. For Model 1 and Model 2, the power of most methods decreased with the MAFs of the disease-associated markers varying from 0.2–0.4. This trend was consistent with the results in Marchini et al. [31]. For Model 3 and Model 4, the power of most methods increased with the MAFs of the disease-associated makers varying from 0.1–0.4. The trend was not obvious for Model 5.

Since ClusterMI grouped high-associativity SNPs into clusters and screened two-locus combinations from these clusters, ClusterMI(E) had a higher power than other approaches on the five models (Model 1–Model 5), except a few cases. In such cases, ClusterMI(E) had lower power than DCHE and EDCF. That was because DCHE and EDCF measured the significance via the chi-square test with a lower degree of freedom than ClusterMI(E), and they could report more interactions in such cases. For all models, when N=4000 with $r^{2}=1$ and MAF = 0.1, ClusterMI(A) and ClusterMI(E) both performed poorly. That was because two SNPs of a significant two-locus combination may be divided into different clusters, which dragged down the power of ClusterMI. ClusterMI(E) performed better than HiSeeker(E); this comparison proves that there was a higher probability of detecting significant two-locus interactions in a high-associativity group and that high-order SNP interactions can be derived from these significant two-locus combinations. Another interesting observation was that the power of EDCF drastically decreased when N=4000 with $r^{2}=0.7$ and MAF = 0.2. One possible reason was that EDCF divided each three-locus combination into three groups and used the chi-square test with two degrees of freedom to measure the significance, resulting in more false positives.

ClusterMI(E) had better performance than DECMDR, HiSeeker(A) and ClusterMI(A), since ClusterMI(E) employed exhaustive search to find optimal solutions without considering the time complexity, while DECMDR, HiSeeker(A) and ClusterMI(A) applied heuristic search. ClusterMI(A), HiSeeker(A) and DECMDR performed poorly when N=2000, which was due to the random nature of these approaches, and the optimal solution was not guaranteed. Although both HiSeeker(A) and ClusterMI(A) utilized the ACO algorithm, in most cases, ClusterMI(A) had relatively superior performance to HiSeeker(A). That was because ClusterMI(A) utilized the conditional mutual information to only screen two-locus combinations in the high-associativity SNP groups and avoided a large number of noisy combinations. In most cases, DECMDR had the lowest power, since it only reported the best solution. When N=4000, ClusterMI(A) had a comparable power to DCHE in most cases. These results demonstrated the effectiveness of ClusterMI in detecting high-order SNP interactions on small datasets.

The high-dimensional simulation dataset with 8000 samples (4000 cases and 4000 controls) and 3000 SNPs for Model 5 was further used to test ClusterMI and other comparison approaches. The parameter settings including MAF and LD were the same as above described. We also used the same simulation program as Zhang et al. [14] to simulate 100 datasets under different parameters settings.

**Figure 4:**
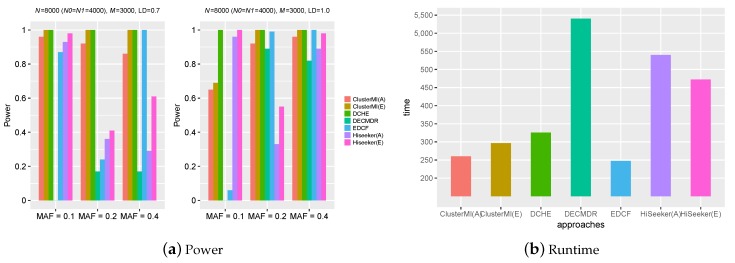
Powers and runtime of different approaches on Model 5 under different allele frequencies (MAF) and linkage disequilibrium (LD) with 8000 samples and 3000 SNPs. (**a**) Power; (**b**) runtime.

Figure 4a reveals the power of different comparison approaches on Model 5. ClusterMI(E) and DCHE obtained the highest power (equal to one) except the case of ClusterMI(E) with $r^{2}=1.0$, MAF = 0.1. In this case, since two SNPs of a significant two-locus combination may be divided into different clusters, ClusterMI performed not so well. This is consistent with the results on the above small simulation datasets. HiSeeker lost its power on high-dimensional simulation datasets when $r^{2}=0.7$, MAF = 0.2 or 0.4 and $r^{2}=1.0$, MAF = 0.2. One possible reason was that HiSeeker employed the chi-square test with seven degrees of freedom to divide all two-locus combinations into two groups, resulting in more false positives with more SNPs. The power of EDCF drastically decreased with $r^{2}=0.7$, MAF = 0.2. This is also consistent with the results on small simulation datasets. Furthermore, EDCF also performed poorly when $r^{2}=1.0$, MAF = 0.1 with high dimensional datasets. The power of DECMDR significantly improved when $r^{2}$ increased from 0.7–1.0, and MAF increased from 0.1–0.2, 0.4. In most cases, ClusterMI(A) outperformed HiSeeker(A) and DECMDR.

Figure 4b reveals the runtime of different comparison approaches on Model 5. ClusterMI was faster than the other approaches except EDCF. In this case, since the clustering process needed some time to reach convergence, ClusterMI was slower than EDCF, but it had a better performance than EDCF. DECMDR employed differential evolution to improve the efficiency, but because of the time-consuming MDR, its runtime still significantly increased on the high-dimensional simulation datasets.

### 2.3. Experiments on Real Data

#### 2.3.1. Experiments on Breast Cancer (BC) Data

It has been reported that breast cancer is caused by a combination of genetic and environmental risk factors [33]. A real breast cancer (BC) dataset collected from WTCCC [29] was used to further evaluate ClusterMI. The BC dataset included a total of 15,347 SNPs, including 1045 cases with breast cancer and 3893 controls. Quality control was performed to exclude the samples and SNPs with a very low call rate, such as an SNP, the call rate of which was <95% across all samples, the *p*-value (Hardy–Weinberg equilibrium) <0.0001 in controls or a sample with a call rate of <98%. Then, SNPs with MAF <0.1 were further excluded. This process produced a BC dataset containing 1045 cases and 3893 controls with 5607 SNPs.

ClusterMI took 10 min to analyze the BC dataset. In the screening stage, ClusterMI employed conditional mutual information combined with the chi-square test following $2^{3}-1$ degrees of freedom to screen the significant two-locus combinations. Since the value of conditional mutual information between two SNPs was generally above 3.7, we set the threshold as 3.76. The threshold of the chi-square was set to $10^{-5}$. Namely, a two-locus combination was considered as significant when its *p*-value was below the chi-square threshold. In this way, 3812 two-locus combinations were retained for the next stage. Many retained two-locus combinations were significant, and several representative ones are reported in Table 1. rs1108842 belongs to gene GNL3 on chromosome 3. The protein encoded by this gene may be associated with stem cell proliferation and may be involved in tumorigenesis. rs879882 is located in gene POU5F1 on chromosome 6. Aberrant expression of POU5F1 in adult tissues leads to tumorigenesis [34]. This gene can participate in a translocation with the Ewing’s sarcoma gene on chromosome 21, which also leads to tumor formation. rs17822931 is located in gene ABCC11 on chromosome 16. It is reported that ABCC11 is highly expressed in aggressive breast cancer subtypes and is associated with poor prognosis [35]. rs3785181 is located in gene GAS11 on chromosome 16. GAS11 is a putative tumor suppressor gene and is reported as being associated with breast cancer [36].

In the search stage, ClusterMI also identified some significant three-locus combinations, and a representative one is reported in the last row of Table 1. The three-locus combination (rs9257694, rs2523608, rs11244) is in the major histocompatibility complex (MHC) region on chromosome 6. rs2523608 is located in gene HLA-*B*, which belongs to HLA class I heavy chain paralogues. Antigen expression of HLA class I is associated with the aggressiveness and prognosis of breast cancer [37]. These significant two-locus combinations and three-locus combinations associated with breast cancer demonstrate the effeteness of ClusterMI in detecting SNP interactions on genome-wide data.

Furthermore, we also ran HiSeeker, EDCF and DCHE on the BC dataset. HiSeeker took 15 min to analyze this dataset, and selected 2576 two-locus combinations in the first stage. SNP interactions identified by HiSeeker were (rs1108842, rs4687657) on chromosome 3, (rs4408545, rs3785181) on chromosome 16 and (rs879882, rs2523608, rs592229) on chromosome 6. rs1108842 in gene GNL3, rs3785181 in gene GAS11, rs879882 in gene POU5F1 and rs2523608 in gene HLA-B are reported to have a high probable association with tumor or breast cancer. All of these SNPs can be identified by ClusterMI. In addition, ClusterMI also identified another SNP interaction (rs17822931, rs3785181) on chromosome 16. rs17822931 is in gene ABCC11, which is reported to be associated with breast cancer. EDCF and DCHE heavily suffered from the marginal effects, and they did not identify interactions associated with breast cancer in the BC dataset.

#### 2.3.2. Experiments on Celiac Disease (CD) Data

Celiac disease (CD) is a common heritable chronic inflammatory condition of the small intestine induced by dietary wheat, rye and barley in susceptible individuals [38]. After the same quality control for the CD dataset as the BC dataset, we selected an SNP subset of the CD dataset containing 5889 SNPs with 3796 controls and 8154 controls.

ClusterMI took 15 min to analyze the CD dataset. In the screening stage, ClusterMI also employed conditional mutual information combined with the chi-square test to screen two-locus combinations. For the threshold of cMI, since the values of conditional mutual information between two SNPs for CD dataset were generally above 2.1, we selected a larger value 2.19 as the cMI threshold. For the threshold of the chi-square, we used the same value as the BC dataset (namely, $10^{-5}$). ClusterMI retained 13,708 two-locus combinations for the next search stage. Hundreds of two-locus combinations were significant; among them, some representative ones are reported in Table 2. rs3748816 is located in gene MMEL1 on chromosome 1. MMEL1 is expressed mainly in testis with weak expression in the brain, kidney and heart. Aberrant expression of this gene may lead to Celiac disease [39]. rs3816281 is located in gene PLEK on chromosome 2. PLEK has biased expression in lymph node and has been evidenced to be able to cause Celiac disease [39]. In the search stage, ClusterMI identified a significant three-locus combination (rs2298428, rs1321, rs5771069) on chromosome 22; among them, rs2298428 is located in gene YDJC. YDJC has ubiquitous in kidney and colon. It is reported that rs2298428/YDJC is associated with Celiac disease [39].

Similarly, we also ran HiSeeker, EDCF and DCHE on the CD dataset. HiSeeker took 40 min to analyze the dataset and selected 11,453 two-locus combinations in the first stage. HiSeeker detected an SNP interaction (rs375555, rs542441) on chromosome 6, in which rs542441 in gene UQCC2 has a high probability of being associated with Celiac disease. Although ClusterMI did not identify rs542441, it detected more interactions associated with Celiac disease. EDCF and DCHE again did not detect SNP interactions associated with Celiac disease on the dataset.

### 2.4. Runtime Analysis

**Figure 5:**
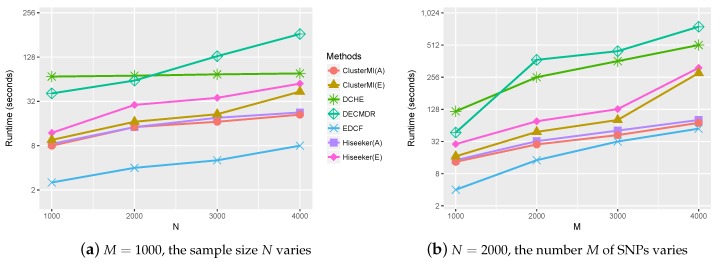
Runtime of different approaches on the simulated datasets. (**a**) The sample size *N* varies from 1000–4000 with the number of SNPs *M* = 1000; (**b**) the number of SNPs *M* varies from 1000–4000 with the sample size N=2000.

Computational efficiency is another key performance index that needs to be considered in high-order SNP interactions’ detection. The runtime of ClusterMI was compared to those of the other four approaches under varying sample sizes *N* and number of SNPs *M*. The recorded runtimes of these approaches are shown in Figure 5. Since EDCF, DCHE, Hiseeker and ClusterMI utilized bitwise computing and stored SNP genotype data in a bitwise data structure, their runtimes were smaller than DECMDR. DECMDR applied the differential evolution algorithm (DE) to improve the efficiency, but the multifactor-dimensionality reduction (MDR) algorithm was still time consuming. Therefore, DECMDR had a greater runtime than the other approaches. HiSeeker(A) was faster than HiSeeker(E), and ClusterMI(A) was faster than ClusterMI(E), since the exhaustive search was time consuming. ClusterMI applied a clustering algorithm to divide all SNPs into several clusters, and it only needed to analyze two-locus combinations in each group, while EDCF, DCHE and Hiseeker exhaustively analyzed all two-locus combinations. Therefore, ClusterMI was faster than DCHE and Hiseeker. Since the clustering process needed some time to reach convergence, ClusterMI was slower than EDCF, but it had better performance than EDCF.

## 3. Materials and Methods

**Figure 6:**
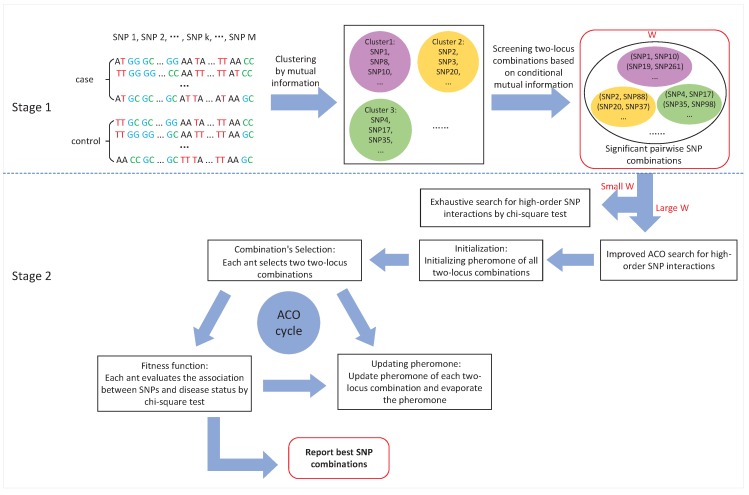
Procedure overview of ClusterMI (Clustering combined with Mutual Information). SNP: single nucleotide polymorphisms; ACO: ant colony optimization algorithm.

Given a genotype dataset consisting of *N* individuals ($N_{0}$ controls, $N_{1}$ cases) and *M* SNPs, we use *y* to denote the phenotype of individuals: y=0 denotes control, and y=1 denotes case; and we use $S_{i}$ (i=1,2,…,M) to denote the *i*-th SNP. In this paper, we mainly focus on the case-control study and assume that all SNPs are biallelic. We suppose that *A* is the major allele and *a* is the minor allele. Each SNP has three genotypes: homozygous reference genotype (AA), heterozygous genotype (Aa) and homozygous variant genotype (aa). Generally, they are coded as {1, 2, 3}, respectively. The whole framework of ClusterMI is illustrated in Figure 6.

ClusterMI is a two-stage approach. In the first stage, it screens candidate two-locus combinations using clustering and mutual information. In the second stage, it detects high-order SNP interactions based on two alternative search strategies (exhaustive search and improved ACO search) for different sizes of candidate set *W*. The following two subsections elaborate on these two stages.

### 3.1. Stage 1: Candidate SNP (Single Nucleotide Polymorphisms) Combinations Selection

We assume that SNPs with high associativity usually have a higher probability of being identified as significant SNP interactions. Given this, ClusterMI clusters SNPs into several groups and then selects two-locus combinations from each group. Since only two-locus combinations in each group need to be analyzed, clustering SNPs can greatly reduce the search space and computation complexity. For a GWAS dataset containing 1 million SNPs, the number of two-locus combinations that need to be analyzed is $5\times 10^{11}$. However, after dividing these SNPs into 100 groups, the number of two-locus combinations can be reduced to $5\times 10^{9}$.

Given a dataset *D* with *N* samples and *M* SNPs, the clustering process is described as follows:

(a) Initialization: *k* SNPs are randomly selected from *M* SNPs as initial centroids of *k* clusters $C_{j}\left(j=1,2,\ldots ,k\right)$, and *k* is the preset number of SNP groups.
(b) Clustering: Mutual information can measure the dependency or associativity between two variables [40,41]. Given this, we take mutual information to measure the associativity between two SNPs. For an SNP pair $\left(S_{i},S_{j}\right)$, the mutual information can be calculated as:
  (3) $$MI\left(S_{i},S_{j}\right)=\sum_{u=1}^{3}\sum_{v=1}^{3}P\left(S_{i}=u,S_{j}=v\right)log\frac{P(S_{i}=u,S_{j}=v)}{P\left(S_{i}=u\right)P\left(S_{j}=v\right)}$$
  where *u* (or *v*) = {1,2,3} denotes the three genotypes of $S_{i}$ (or $S_{j}$); $P(S_{i}=u,S_{j}=v)$ denotes the joint probability of $S_{i}$ and $S_{j}$; $P(S_{i}=u)$ and $P(S_{j}=v)$ are the marginal probability of $S_{i}$ and $S_{j}$, respectively. For $S_{i}$ and the centroid of cluster $C_{m}\left(m=1,2,\ldots ,k\right)$, ClusterMI calculates the mutual information $MI(S_{i},C_{m})$ between $S_{i}$ and $C_{m}$. $S_{i}$ is divided into the *m*-th (1≤m≤k) group when $MI\left(S_{i},C_{m}\right)>MI\left(S_{i},C_{m^{\prime }}\right)\left(\forall m^{\prime }\neq m\right)$.
(c) Update centroids: In each iteration, ClusterMI updates each centroid after each SNP has been divided into one of the *k* clusters. Suppose $G_{m}$ (1≤m≤k) stores the SNPs of the *m*-th cluster; ClusterMI measures the sum of mutual information of centroid $C_{m}$ as:
  (4) $$sMI\left(C_{m}\right)=\sum_{S_{i}\in G_{m}}MI\left(C_{m},S_{i}\right)$$
  Then, for each SNP $S_{r}\in G_{m}$, ClusterMI also calculates the sum of mutual information of $S_{r}$ in $G_{m}$ as:
  (5) $$sMI\left(S_{r}\right)=\sum_{S_{i}\in G_{m}}MI\left(S_{r},S_{i}\right)$$
  If $sMI\left(C_{m}\right)/sMI\left(S_{r}\right)\leq 1$, ClusterMI renews the centroid of $G_{m}$ as $S_{r}$. For each group, the same procedure is conducted to update the clustering centroid.

Steps (b) and (c) are repeated until the centroids of *k* clusters no longer change or the maximum number of repetitions is reached. In this way, ClusterMI obtains *k* SNP groups. Each cluster has a disjoint subset of all the SNPs, and two-locus interactions with high-associativity SNPs are more likely to be significant and placed into the same subset. As such, ClusterMI can efficiently screen significant pairwise SNP combinations from a reduced number of SNP combinations, instead from all SNP combinations.

After dividing all SNPs into *k* clusters, ClusterMI then applies conditional mutual information to screen two-locus combinations in each cluster as follows:

(a) For the *m*-th cluster, the association between a two-locus combination and the disease can be measured by conditional mutual information [40,41]. The conditional mutual information of a two-locus combination $\left(S_{i},S_{j}\right)\left(S_{i},S_{j}\in G_{m}\right)$ under case (*y* = 1) can be calculated as:
  (6) $$cMI\left(S_{i},S_{j}\right)=\sum_{u=1}^{3}\sum_{v=1}^{3}P\left(S_{i}=u,S_{j}=v|y=1\right)log\frac{P(S_{i}=u,S_{j}=v|y=1)}{P\left(S_{i}=u|y=1\right)P\left(S_{j}=v|y=1\right)}$$
  where $P(S_{i}=u,S_{j}=v|y=1)$ denotes the joint probability of $S_{i}$ and $S_{j}$ under the case; $P(S_{i}=u|y=1)$ and $P(S_{j}=v|y=1)$ are the marginal probability of $S_{i}$ and $S_{j}$ under the case, respectively.
(b) ClusterMI takes a two-locus combination $\left(S_{i},S_{j}\right)$ with $cMI(S_{i},S_{j})>\tau$ as a significant SNP combination, and τ is a user-specific threshold.

For all two-locus combinations in each cluster, ClusterMI applies the same two-step procedure (a) and (b) described above to screen significant SNP combinations. Next, it places all the significant two-locus combinations of each cluster into the candidate set *W*.

### 3.2. Stage 2: High-Order SNP Interactions Detection

In the search stage, ClusterMI provides two alternative strategies (exhaustive search and improved ACO search) to search high-order SNP interactions based on the size of *W*.

#### 3.2.1. Exhaustive Search for a Small Candidate Set (Small *W*)

Exhaustive search is affordable when the significant candidate set *W* is small, and it has a larger chance to detect high-order SNP interactions than heuristic search. To exhaustively search *K*-SNP (K≥3) interactions, ClusterMI combines all candidate SNPs into a set of *K*-SNP, and computes the corresponding *p*-value obtained by $\chi^{2}$-test. ClusterMI reports these *K*-SNP combinations, the *p*-values of which are smaller than a Bonferroni-corrected significance threshold α. Given a preset significance level $\alpha_{0}$, α can be calculated as:

(7) $$\alpha =\alpha_{0}/C_{M}^{K}$$

#### 3.2.2. Heuristic Search for a Large Candidate Set (Large *W*)

When the cardinal of *W* is very large, it is very time consuming or even infeasible to search high-order SNP interactions exhaustively. For a large candidate set, ClusterMI employs an improved ant colony optimization (ACO) algorithm [28] to search high-order SNP interactions in a more efficient way. The ACO search strategy has been widely applied in GWAS studies [27,28,30,42,43,44]. The search space is composed of significant two-locus combinations of *W* obtained in the first stage. Here, we take K=3 as an example to illustrate the ACO search strategy for detecting *K*-SNP (K≥3) interactions as follows:

(i) Initialization: The pheromone value of all two-locus combinations in *W* is initialized as $\gamma_{0}$, which means the association between a combination and disease is treated with equal possibility.
(ii) Combinations selection: ACO introduces n(n<|W|) ants to select two-locus combinations. An ant respectively chooses two combinations as its targeted combination set when K=3. The probability ($P_{a}^{i}\left(t\right)$) for an ant a(0≤a≤n) to select the *i*-th two-locus combination $L_{i}$ at iteration *t* can be defined as [28]:
  (8) $$P_{a}^{i}\left(t\right)=\left\{\begin{array}{c} R\;\;\;\;\;\;\;\;\;\;\;q\leq q_{0}\\ T\;\;\;\;\;\;\;\;\;\;\;q>q_{0} \end{array}\right.$$
  where *q* is a randomly-generated number with a uniform distribution in (0, 1) and $q_{0}\in \left[0,1\right]$ is a user-defined threshold to control the rate of convergence and to avoid falling into the local optimal solution. *R* and *T* can be described as:
  (9) $$R=\left\{\begin{array}{c} \frac{\gamma_{i}{\left(t\right)}^{\alpha }\eta_{i}^{\beta }}{\sum_{j\in W_{a}\left(t\right)}\gamma_{j}{\left(t\right)}^{\alpha }\eta_{j}^{\beta }}\;\;\;\;\;\;\;\;\;\;\;\;\;\;L_{i}\in W_{a}\left(t\right)\\ 0\;\;\;\;\;\;\;\;\;\;\;\;\;\;\;\;\;\;\;\;\;\;otherwise \end{array}\right.$$
  (10) $$T=\left\{\begin{array}{c} 1\;\;\;\;\;\;\;\;\;\;\;\;\;\;\;\;\;\;L_{i}=rand\left(W_{a}\left(t\right)\right)\\ 0\;\;\;\;\;\;\;\;\;\;\;\;\;\;\;\;\;\;\;\;\;otherwise \end{array}\right.$$
  where $\gamma_{i}\left(t\right)$ is the pheromone of the *i*-th two-locus combination $L_{i}$ at iteration *t* and $\eta_{i}$ is the heuristic information. α and β are the weight parameters of pheromone and heuristic information. ClusterMI sets η, α and β as 1, indicating that each two-locus combination is treated equally before the optimization phase. $W_{a}\left(t\right)$ is a set of two-locus combinations that are not selected by ant *a* at iteration *t*, and $rand(W_{a}\left(t\right))$ denotes that the ant *a* randomly selects a two-locus combination from $W_{a}\left(t\right)$.
(iii) Evaluation: To search high-order SNP interactions, two selected combinations of each ant are merged into a new three-locus combination $L^{{}^{\prime }}$: $\left(S_{i},S_{j},S_{k}\right)$. The fitness value of $\left(S_{i},S_{j},S_{k}\right)$ is calculated by the $\chi^{2}$-test:
  (11) $$\chi^{2}\left(S_{i},S_{j},S_{k}\right)=\sum_{u=1}^{3^{3}}\sum_{v=0}^{1}\frac{{\left(N_{uv}-N_{u+}N_{+v}/N\right)}^{2}}{N_{u+}N_{+v}/N}$$
  where $\chi^{2}\left(S_{i},S_{j},S_{k}\right)$ follows a $\chi^{2}$-test with $3^{3}-1$ degrees of freedom, $N_{uv}$ means the number of samples with the *u*-th joint genotype for the three-locus combination and the *v*-th disease status, $N_{u+}$ means the number of samples with the *u*-th joint genotype under the case (y=1) and control (y=0) and $N_{+v}$ means the number of samples with the *v*-th disease status for all joint genotypes.
(iv) Pheromone update: In each iteration, each ant selects two two-locus combinations, the corresponding pheromone of each combination is updated as:
  (12) $$\gamma_{i}\left(t+1\right)=\left(1-\rho \right)\gamma_{i}\left(t\right)+\Delta \gamma_{i}\left(t\right)$$
  where ρ is the evaporating coefficient and $\Delta \gamma_{i}$ is the variation of pheromones of the *i*-th two-locus combination $L_{i}$; it is updated as
  (13) $$\Delta \gamma_{i}\left(t\right)=\sum_{a=1}^{n}\Delta \gamma_{i}^{a}\left(t\right)$$
  (14) $$\Delta \gamma_{i}^{a}\left(t\right)=\left\{\begin{array}{c} \chi^{2}\left(L^{{}^{\prime }}\right)\;\;\;\;\;\;\;\;\;\;\;a\in A_{i}\left(t\right)\\ 0\;\;\;\;\;\;\;\;\;\;\;\;\;\;\;\;\;\;otherwise \end{array}\right.$$
  where $A_{i}\left(t\right)$ is a set of ants that select the *i*-th two-locus combination $L_{i}$ at iteration *t*, $L^{{}^{\prime }}$ is a three-locus combination identified by ant *a* at iteration *t* and $\chi^{2}\left(L^{{}^{\prime }}\right)$ is the fitness value of $L^{{}^{\prime }}$.

The improved ACO algorithm introduces a memory strategy to save the optimal solutions in each iteration and speed up the convergence [30]. The above three iterative operations (Steps (ii)–(iv)) are repeated until a preset number of iterations is reached. After applying the improved ACO algorithm, the merged SNP combinations with the highest $\chi^{2}$ statistics are reported. Next, ClusterMI re-applies the $\chi^{2}$-test to measure the *K*-SNP combinations of all these reported combinations. It finally takes *K*-SNP subsets, the *p*-values of which are below the Bonferroni-corrected significance level $\alpha_{0}/C_{M}^{K}$, as the detected *K*-SNP interactions.

## 4. Conclusions

In this paper, we developed a two-stage approach called ClusterMI to detect high-order SNP interactions from genome-wide case-control data. Considering SNP groups with high associativity usually have a higher probability to encompass high-order SNP interactions and the high computational complexity with the exponential growth of high-order SNP combinations, ClusterMI employs a clustering algorithm that utilizes mutual information as the association measurement, to divide all SNPs into several clusters. Next, it applies conditional mutual information to screen significant two-locus combinations in each group. As such, ClusterMI avoids exhaustively analyzing all two-locus combinations and greatly reduces the computational complexity. In the search stage, ClusterMI provides two alternative search strategies (exhaustive search and improved ACO search) to detect high-order SNP interactions based on the size of candidate two-locus combinations. Exhaustive search is used for a small candidate set, and improved ant colony optimization (ACO) is used for a large candidate set. Extensive simulation experiments compared to representative approaches show that ClusterMI has a better performance. Experiments on two real case-control datasets also demonstrate that ClusterMI can identify high-order SNP interactions from genome-wide data.

ClusterMI can be an effective methods for detecting high-order interactions, and its main contributions are:

- The clustering algorithm utilized in the screening stage of ClusterMI can place SNPs with high-associativity into a cluster, in which the true SNP interactions can be more easily identified. In addition, it greatly reduces the computational complex by avoiding analyzing the whole set of two-locus combinations.
- The conditional mutual information-based evaluation strategy in each high-associativity cluster can effectively screen two-locus combinations and reduce the search space of ACO; it also can improve the power of SNP interactions’ detection and make high-order SNP interactions’ detection on genome-wide data more efficient.

Although ClusterMI shows good performance on both simulated and real datasets, it still has some limitations and can be improved. In the future work, we will investigate more efficient and effective clustering algorithms [45,46,47], distance metrics [48,49] and advanced evolutionary algorithms [50,51,52,53] to further improve the effectiveness and efficiency of ClusterMI. The identified SNP interactions are still limited, and we will investigate data integrative solutions to explore SNP interactions specific to known disease pathways and to detect more SNP interactions.

## Figures and Tables

**Table 1:** Significant two-locus and three-locus combinations identified by ClusterMI on the Wellcome Trust Case Control Consortium (WTCCC) breast cancer (BC) data.

Chromosome	SNP Combinations	Related Genes	Single-Locus *p*-Value	Combination *p*-Value
chr3	(rs13100173, rs1108842)	(HYAL3, GNL3)	($4.856\times 10^{-2},8.422\times 10^{-1}$)	$2.241\times 10^{-8}$
chr6	(rs9257694, rs879882)	(LOC105375005, POU5F1)	($1.370\times 10^{-2},1.135\times 10^{-1}$)	$3.682\times 10^{-6}$
chr6	(rs3094576, rs644827)	(*, SLC44A4)	($1.129\times 10^{-1},1.999\times 10^{-4}$)	$8.598\times 10^{-15}$
chr16	(rs17822931, rs3785181)	(ABCC11, GAS11)	($1.778\times 10^{-1},9.371\times 10^{-1}$)	$8.462\times 10^{-6}$
chr16	(rs7190823, rs4408545)	(FANCA, AFG3L1P)	($1.772\times 10^{-1},1.410\times 10^{-1}$)	$1.240\times 10^{-13}$
chr6	(rs9257694, rs2523608, rs11244)	(LOC105375005, HLA-B, HLA-DOB)	($1.370\times 10^{-2},3.239\times 10^{-1},3.289\times 10^{-1}$)	$5.551\times 10^{-16}$

* Indicates that the related gene is unknown. *p*-value is estimated by the chi-square test.

**Table 2:** Significant two-locus and three-locus combinations identified by ClusterMI on the WTCCC celiac disease (CD) data.

Chromosome	SNP Combinations	Related Genes	Single-Locus *p*-Value	Combination *p*-Value
chr1	(rs3748816, rs3795263)	(MMEL1, ACTRT2)	($6.691\times 10^{-3},4.345\times 10^{-1}$)	$1.096\times 10^{-8}$
chr2	(rs3816281, rs4973588)	(PLEK, NGEF)	($1.231\times 10^{-2},8.152\times 10^{-2}$)	$7.457\times 10^{-6}$
chr6	(rs3823418, rs4151664)	(PSORS1C1, NELFE)	($1.279\times 10^{-3},2.512\times 10^{-3}$)	$5.807\times 10^{-10}$
chr6	(rs2021723, rs3093662)	(TRIM40, TNF)	($2.711\times 10^{-1},1.033\times 10^{-3}$)	$6.297\times 10^{-8}$
chr22	(rs2298428, rs1321, rs5771069)	(YDJC, ALG12, IL17REL)	($9.164\times 10^{-3},2.301\times 10^{-1},8.773\times 10^{-3}$)	$5.551\times 10^{-13}$

*p*-value was estimated by the chi-square test.
